# Physicochemical Characterisation of Seeds, Oil and Defatted Cake of Three Hempseed Varieties Cultivated in Spain

**DOI:** 10.3390/foods13040531

**Published:** 2024-02-09

**Authors:** Rito J. Mendoza-Pérez, Grazielle Náthia-Neves, Beatriz Blanco, Antonio J. Vela, Pedro A. Caballero, Felicidad Ronda

**Affiliations:** 1 Department of Agriculture and Forestry Engineering, Food Technology, College of Agricultural and Forestry Engineering, University of Valladolid, 34004 Palencia, Spain; ritojose.mendoza@uva.es (R.J.M.-P.); graziellenathia.neves@uva.es (G.N.-N.); avelacor@purdue.edu (A.J.V.); mfronda@uva.es (F.R.); 2 Research Institute on Bioeconomy - BioEcoUVa, PROCEREALtech Group, University of Valladolid, 47011 Valladolid, Spain; 3 Chemical Engineering Section, Department of Biotechnology and Food Science, Faculty of Sciences, University of Burgos, 09001 Burgos, Spain; bblanco@ubu.es; 4 Whistler Center for Carbohydrate Research, Department of Food Science, Purdue University, West Lafayette, IN 47907-2053, USA

**Keywords:** partially defatted hemp flour, hemp by-product, hempseeds, functional properties, DSC

## Abstract

The increasing use of hempseed in food products highlights the need for a comprehensive database for scientific research and industrial applications. In food development, information about the techno-functional properties of raw materials plays a crucial role in determining the suitability of each product for specific applications. Thus, this study aims to characterise three hempseed varieties (*Ferimon*, *Henola* and *Uso-31*), comparing their physicochemical and nutritional compositions. Moreover, the study investigates the impact of hempseed varieties on the techno-functional, physical and thermal properties of the partially defatted hempseed flours (PDHFs) obtained from single screw pressing (SSP) oil extraction. The fatty acid and tocopherol profiles of the dehulled seeds and oil were also analysed. Significant variations in yield and physical properties were observed among hempseed varieties, influenced by genetics, adaptation to agro-climatic conditions and cultivation systems. Despite its lower yield (kg/ha), *Uso-31* exhibited superior 1000-seed weight, dehulling yield and larger mean seed size (1.79 ± 0.02 mm). Hempseed oil was rich in unsaturated fatty acids, particularly linoleic (51.2–53.4 g/100 g oil) and α-linolenic (14.88–18.97 g/100 oil) acids, showing variations in γ- and α-tocopherols depending on the variety. The variety also influenced the least gelation concentration (LGC) and techno-functional properties such as water absorption capacity (WAC), emulsifying activity (EA) and emulsion stability (ES). SDS-PAGE and DSC measurements indicated the presence of 11S and 7S globulin proteins with denaturation temperatures above 87.8 °C. These findings confirm that the studied hempseed flours are valuable techno-functional and nutritional ingredients suitable for sustainable food formulations.

## 1. Introduction

Hemp (*Cannabis sativa* L.) is a versatile plant with applications in textile, food and nutraceutical purposes [1], and it is widely cultivated for commercial use in Europe, China, Japan and the USA [2]. In the European Union (EU), there are currently 75 approved varieties of hemp for industrial use [3], and cultivation has experienced a remarkable boom, with a 75% increase in the cultivated area from 2015 to 2019, reaching a considerable 34,960 hectares [3]. Despite this growth, hemp constitutes only 0.02% of the total cultivated area in the EU [3]. Hemp cultivation requires special permits, which are conditional on the use of varieties authorised by EU regulations. 

For cultivars primarily used for fibre production, the seeds are often considered a product of minor interest [4]. However, several authors have reported the substantial nutritional value of industrial non-dehulled hempseeds, generally composed of 30–35 g/100 g of lipids, 20–25 g/100 g of protein (highly digestible protein, making them well-suited for both human and animal consumption), 30–35 g/100 g of dietary fibre and a rich array of minerals [4,5,6,7,8]. Complementarily, previous studies have reported that the composition of hempseeds is influenced by the specific variety under investigation [8,9,10,11], as well as by the dehulling process. To our knowledge, there are no studies regarding the comparative dehulling efficiency of hempseed varieties. House et al. 2010 [12] reported a composition of dehulled hempseeds with mean values of 46.7 g/100 g crude oil and 35 g/100 g crude protein, varying their nutritional profile compared to the profile of non-dehulled hempseeds and the potential applicability of their components, particularly proteins, in the food industry [13]. As a result, dehulled hempseed is commonly offered as commercial food for human consumption [14], and there is a growing interest among EU consumers in incorporating these seeds into their diets [3]. 

Hempseeds are mainly used for oil extraction [15] due to their quantity and richness in essential fatty acids, consisting of remarkable amounts of linoleic acid (omega-6) and α-linolenic acid (omega-3), often present in a favourable 3:1 ratio [5,6]. Different methods for hemp oil extraction have been studied, including pressure extraction systems [12,15]. The use of screw presses has emerged as an optimal option, preserving the original properties of the raw material, achieving high yields at low costs and producing oils of a suitable quality [16]. The scientific literature extensively reports on the hempseed oil obtained by press extraction systems [1,17,18]. Hempseed oil extraction generates a by-product known as cake, traditionally considered as a residue from the oil extraction process. Recent studies have characterised the physicochemical and biochemical properties of the different fractions obtained from the extraction process of non-dehulled hempseed oil, reporting their content of bioactive compounds and antioxidant capacity [9]. Additionally, the amino acid profile and digestibility of the protein fraction from these by-products obtained from non-dehulled seeds have been studied [12]. These studies have demonstrated the high potential of the by-product from non-dehulled hempseed oil extraction for use as a protein-rich food ingredient. However, although there are existing studies addressing the functional properties of hemp protein isolates [19,20], the functional characteristics of the cake obtained after oil extraction have not been investigated yet. Furthermore, there is a lack of information in the current scientific literature regarding how the hemp variety influences the techno-functional characteristics of the partially defatted hempseed flour (PDHF) obtained from it as a cake. Understanding the hydration, emulsifying and foaming properties of PDHF, depending on the variety, is essential to determine its suitability as an ingredient for specific applications in food product development.

Therefore, the aim of this study was to investigate the techno-functional properties of PDHF and assess how these properties are influenced by hemp variety, also addressing the analysis of the characteristics of the dehulled hempseed and the oil obtained, with a particular interest in their nutritional properties. By assessing the techno-functional characteristics of different hempseed varieties and comprehending the synergies among their different components (fibre, fat, ash and proteins), this study aims not only to advance the development of high-quality foods but also to address sustainability concerns. The results from this work will provide the basis for a better selection of hempseed varieties, considering their potential for the utilisation of oil extraction by-products and incorporating them into the production of ingredients with high nutritional value and suitable properties for food production. This will contribute to the valorisation of by-products and increase the added value of bio-waste derivatives, which could play a crucial role in the sustainability of food systems. This study will also make a significant contribution to the existing literature by addressing the efficiency of dehulling hempseeds based on the variety, an aspect that has not been explored previously.

## 2. Materials and Methods

### 2.1. Hempseeds

The hempseed varieties studied in this work were *Ferimon*, *Henola* and *Uso-31*, originating from France, Poland and Ukraine, respectively. These varieties are authorised for cultivation in Spain in accordance with the Royal Decree 17/29/1999 of 12 November 1999 [3,21] and are listed in the EU Common Catalogue of Agricultural Plant Species [22] with a THC content of less than 0.2%. These varieties were cultivated in a field trial located in “Calzada de los Molinos” (42°19′38″ N, 4°39′0″ W, height 824 m above sea level) within the region of Castilla y León, Spain, under ecological and rainfed conditions. An agricultural county was selected where there is particular interest in introducing new industrial crops into the rotation. The soil texture of the experimental plot was sandy loam. The field experiment was conducted from April to October 2020, with a total cumulative rainfall in the period of 169.4 mm. The seeds were obtained by mechanical harvesting after a season characterised by normal temperatures for the area. The seeds, along with their yield data, were kindly provided by Castilla Bio Lab (Palencia, Spain). The seeds were stored in controlled conditions at 4 °C before processing and analysis. 

### 2.2. Dehulling and Physical Characterisation

**Figure 1:**
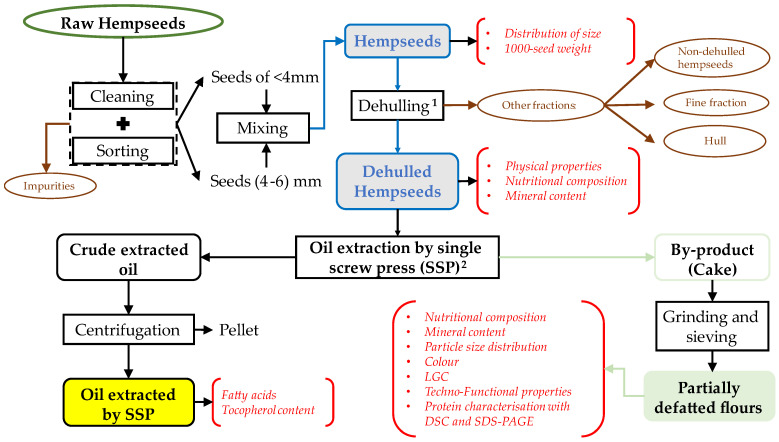
Schematic representation of the processing carried out on hempseeds. The red letters correspond to the analysed parameters and identify the matrices studied. The steps marked with superindices 1 and 2 represent the steps where yields and losses were quantified and reported.

Figure 1 illustrates the schematic representations of the processing carried out on hempseeds from the experimental crop field studied in this work.

First, the provided hempseeds were cleaned to remove impurities and were subsequently sorted based on size using a grain-cleaning and sorting machine (Tripette & Renaud E.I., Asnières-sur-Seine, France). This sorting procedure allowed the establishment of the seed size distribution, resulting in two distinct categories: seeds smaller than 4 mm and seeds measuring between 4 and 6 mm. The fractions were weighted, and the seed size distribution was determined. Seeds from both size categories were mixed in a 1 L polytetrafluoroethylene (PTFE) cylinder tube connected to a rotary homogeniser operating at 70 rpm for 10 min to obtain a homogeneous sample. Then, the count of 1000 seeds was conducted employing an electronic grain counter (CHOPIN Technologies, Villeneuve-La-Garenne, France). The 1000-seed weight was measured using a TXB precision scale (COBOS, Barcelona, Spain). These measurements were carried out in triplicate.

Next, hempseeds were dehulled using a laboratory-scale disc mill (KoMo Gmbh & Co. KG, Munich, Germany) and subsequently passed through a sorting machine (Tripette & Renaud E.I., Asnières-sur-Seine, France) to obtain the dehulled hempseeds (≤3.25 mm) and the other fractions (fine fraction and non-dehulled seeds). The non-dehulled seeds were manually separated, and the loss was determined by the difference between the raw material used in the dehulling process and previous fractions. For this study, the dehulled hempseeds were used for characterisation, and all fractions resulting from the dehulling process were quantified to determine the process yield. Both the non-dehulled and dehulled seeds were stored at 4 °C until further use. The cleaning–sorting and dehulling process were conducted using hempseeds at room temperature.

In the dehulled seeds, the following physical properties were determined: granulometry, bulk density (kg/m^3^) and true density (kg/m^3^). Granulometry was measured using an electromagnetic sieve shaker (Cisa Cedaceria Industrial, S.L., Barcelona, Spain) following the AACC official method 55–60.01 (AACC, 2011) with some modifications. A 150 g sample was sieved using a sieve array (2.80, 2.36, 2.00, 1.70 and 1.40 mm) with 5 min of shaking, and the percentage fraction of the sample retained on each sieve was quantified through weighing. The average seed size (a_50_) and the coefficient of variation (CV) were determined using the Rens method described by Argaw (2007) [23]. Bulk density was determined with a tared 100 mL graduated cylinder according to the methods described by Solaesa et al. (2020) [24]. The true density (TD) was determined by the liquid displacement method employing toluene, following the procedure described by Abebe et al. (2015) [25] and using 50 mL pycnometers for the determination. Granulometry, bulk density and true density were measured at least in duplicate. 

### 2.3. Dehulled Hempseed Processing

Hempseed oil was obtained by pressing 200 g of dehulled hempseeds using a single screw press (SSP) (Cgoldenwall CZR 309, Hangzhou, China) with a length of 194 mm and diameter of 20 mm. This product was identified in Figure 1 as oil extracted by SSP. Each extraction was performed for 3 min, during which the temperature (not exceeding 80 °C) was monitored using a Testo 735-2 digital thermometer (Instrumentos Testo S. A., Barcelona, Spain). Both extraction and temperature measurements were performed in triplicate. Hemp oil was then centrifuged (7000× *g*, 30 min, 10 °C) to remove any remaining solids (Thermo Fisher Scientific, Waltham, MA, USA), referred to as a pellet in Figure 1. The extracted oil was stored in dark glass bottles at 5 °C until further analysis. The oil extraction yield was calculated as g of oil/100 g of dehulled seeds. The oil content of the dehulled hempseeds was determined by the Soxhlet method 30-25.01 [26], and the percentage of the oil recovered by SSP was calculated as the weight ratio of the extracted oil to the oil content of the sample. The cake obtained as a by-product from the oil extraction process was ground using an electric coffee grinder (Cgoldenwall HC-400, Hangzhou, China) and sieved through a 250 μm sieve. The time elapsed from the reception of raw hempseeds to the oil extraction process did not exceed 15 days. The partially defatted hempseed flour (PDHF) samples were stored at 4 °C until used.

### 2.4. Nutritional Composition

The nutritional compositions of both dehulled hempseeds and the partially defatted flours were determined. Moisture content was measured following the Official AACC Method 44-19 [27]. Total nitrogen (N) was quantified by an automatic combustion method using a LECO CNS 928 carbon, nitrogen and sulfur analyser (LECO Instrumentos S.L., Madrid, Spain). The total protein content was calculated using a conversion factor of 6.25. Total fat was determined according to the Official AACC Method 30-25.01 [26], using petroleum ether as the extraction solvent at 60 °C for 5 h at a condensation rate of 5–6 drops/s, with 25 cycles. Fat content was calculated based on the weight of the collected oil and expressed as crude fat in the sample (g/100 g). Ash content was determined by the incineration method UNE-EN ISO 2171 [28]. Fibre content was determined using the AOAC 991.43 official method [29]. All determinations were performed in duplicate. 

The mineral content was determined using the method described by Ronda et al. (2015) [30] on aliquots (~0.7 g) of dehulled seeds and partially defatted flours digested with 8 mL of high purity 65% HNO_3_ and 2 mL of 30% H_2_O_2_ using microwave technology (ETHOS SEL, Milestone, Italy). To analyse the mineral composition, a radial simultaneous inductively coupled plasma optical emission spectrometry (ICP-OES) Varian 725 ES spectrophotometer (Agilent Technologies, Santa Clara, CA, USA) was employed for the determination of P, Mg, K, Ca, Na, Fe, Zn, Mn and Cu. Additionally, a mass spectrophotometer (ICP-MS) Agilent 7800 (Agilent Technologies, Santa Clara, CA, USA) was used for the determination of Co and Se. 

### 2.5. Characterisation of the Extracted Oil

The fatty acid (FA) profile and tocopherols content were analysed in both the dehulled hempseeds (from which oil was extracted by the Soxhlet method) and in the oil extracted by SSP. The FA profile was determined according to the AOAC official method 41.1.30 [31], using an Agilent-6890N gas chromatograph (Agilent Technologies, Santa Clara, CA, USA) equipped with a CP-SIL88 Column (100 m × 0.2 mm i.d. × 0.36 μm) (Agilent Technologies, Santa Clara, CA, USA) following the method described by Rebolleda et al. (2012) [32]. An internal standard quantification method was applied using FA chromatographic standards (Sigma-Aldrich Inc., St. Louis, MO, USA) and methyl tricosanoate as the internal standard. 

The tocopherols content (mg/g) was determined according to the IUPAC 2.432 official method (IUPAC, 1992), following the procedure reported by Rebolleda et al., 2012 [32]. An Agilent HPLC series 1100 (Agilent Technologies, Santa Clara, CA, USA) with ACE 5 silica column (250 mm × 4.6 mm) was used for the analysis. The eluent consisted of n-hexane/2-propanol, flowing at a rate of 0.8 mL/min. Individual compounds of α-, β-, γ- and δ-tocopherols were identified and quantified using an external calibration curve of the corresponding standard compounds. The determination of fatty acids and tocopherols content was made at least in duplicate.

### 2.6. Characterisation of the Partially Defatted Hempseed Flour (PDHF) 

Particle size distribution, colour coordinates, and the least gelation concentration (LGC) were determined in the partially defatted hempseed flour. The proteins’ molecular weight distribution, techno-functional and thermal properties were measured on totally defatted samples obtained from PDHF by fat solvent extraction (Soxhlet method). 

#### 2.6.1. Particle Size Distribution

The particle size distribution was determined using a Mastersizer 2000 laser diffraction particle size analyser (Malvern Instruments Ltd., Malvern, UK) coupled with a Sirocco dry powder feeder. The reported results include the median diameter (D_50_) and the dispersion measurement [(D_90_ − D_10_)/D_50_], as described in [33]. Measurements were performed in triplicate.

#### 2.6.2. Colour

Colour measurements were carried out using a colourimeter PCE-CSM5 (PCE Instruments, Meschede, Germany) and CQCS (version 3.0) software. Results were obtained in the CIE L* a* b* and CIE L* C* h coordinates using a D65 standard illuminant and a 10° standard observer. The hue (h) and the chroma (C*) were calculated as described by Abebbe et al. (2015) [25]. The reported values included L* (lightness from 0: black to 100: white), hue (h) [red (h = 0), yellow (h = 90), green (h = 180) and blue (h = 270)] and Chroma (C*). Measurements were made in triplicate.

#### 2.6.3. Least Gelation Concentration (LGC)

LGC was determined using the method described by Solaesa et al. (2020) [24] with some modifications. The suspensions were prepared in test tubes by dispersing hempseed flour at concentrations ranging from 2 to 20% (*w*/*v*) in 5 mL of distilled water and heating them at 90 °C for 1 h in a water bath. Then, the tubes containing the samples were cooled under tap water and left to stand for 3 h at 15 ± 2 °C. The LGC was determined as the concentration at which the sample did not slip when inverting the tube containing the gel. Samples were evaluated at least in duplicate.

#### 2.6.4. Techno-Functional Properties

Water absorption capacity (WAC), water absorption index (WAI), water solubility index (WSI) and swelling power (SP) of the totally defatted hempseed flours were determined following the method described in Calix-Rivera et al. (2023) [34]. Foaming capacity (FC) and foam stability (FS) were analysed according to the procedures described by Abebe et al. (2015) [25]. Emulsifying activity (EA) and emulsion stability (ES) were determined according to the method described by Solaesa et al. (2020) [24] with some modifications. The flour (7 g) was mixed with 100 mL of water and 100 mL of corn oil (Koipe Asua, Cordoba, Spain). The mixture was homogenised for 60 s at 1000 rpm using a homogeniser and then evenly divided into 50 mL centrifuge tubes. The initial volume of the emulsion V_1_ was recorded, and then the tubes were centrifuged at 1300× *g* for 5 min, and the volume of the remaining emulsified layer was measured (V_2_). EA was calculated as the V_2_/V_1_ ratio and expressed as a percentage. To determine ES, the tubes containing the emulsions were heated at 80 °C for 30 min, cooled down to room temperature and centrifuged at 1300× *g* for 5 min. ES was calculated as the percentage of emulsion remaining after this process. All properties were determined in triplicate, and the results were referred to flour dry matter (d.m).

#### 2.6.5. Protein Characterisation of Hempseed Flours

The thermal properties of hemp flours were determined using a differential scanning calorimeter DSC3 (Mettler Toledo, Barcelona, Spain) according to the method described by Vela et al. (2021) [33]. A sample of ~6 mg was weighed into a 40 μL aluminium pan, and distilled water was added to reach a flour concentration of ~40% *w*/*w*. The pans were sealed and allowed to equilibrate for 30 min at room temperature before measurement. Measurements were performed from 0 to 115 °C at a heating rate of 5 °C/min, using an empty sealed pan as a reference. Onset (T_O_), peak (T_P_) and endset (T_E_) temperatures (°C) and enthalpy (ΔH) (J/g flour d.m) of denaturation were quantified. Samples were measured at least in duplicate. 

The SDS-PAGE was performed according to the procedure described by Náthia-Neves et al. (2023) [35], with some modifications. Samples were submitted to extraction with protein loading buffer, both with and without 2-mercaptoethanol, at a temperature of 6 °C overnight. The sample concentration in the buffer was 5 mg/mL. After extractions, samples were boiled at 100 °C for 5 min before being loaded into the wells. The protein fractions were resolved using a 12% separating gel and 5% stacking gel. The same amount of protein (35 μg) was loaded into each well under both reducing (with 2-mercaptoethanol) and non-reducing (without 2-mercaptoethanol) conditions. After electrophoresis, protein bands were visualised by staining with 0.1% (*w*/*v*) Coomassie Blue R-250 (Sigma Aldrich, Germany) in a methanol/acetic acid/water (40:10:50, volume) (Merck) and subsequently distained in the solvent mixture. The molecular weight of prominent bands was estimated by comparing them to the NZYBlue Protein Marker (NZytech, Lisbon, Portugal), a mixture of 11 highly purified pre-stained proteins ranging from 10 kDa to 180 kDa. SDS-PAGE electropherograms were generated using a Gel Doc™ EZ Imager (Bio-Rad, Mississauga, ON, Canada) and analysed with Image Lab (version 4.1, Bio-Rad). The relative protein content for the major bands (subunit or protein band) was semi-quantified following the procedure described by Wang et al. (2008) [36] based on the relative area of each band in relation to the total area. Samples were measured in duplicate.

### 2.7. Statistical Analysis

The obtained results were statistically analysed using Statgraphics Centurion XIX software (Bitstream, Cambridge, MN, USA). Analysis of variance (ANOVA) and the least significant difference (LSD) test at *p*-value ≤ 0.05 were performed.

## 3. Results and Discussion 

### 3.1. Agronomic Yield of Hemp Varieties and the Seed Dehulling Process Yield

The yield of seeds (in kg/ha), size distribution (%) and their 1000-seed weights are shown in Table 1. The results indicate that *Uso-31* significantly differed from the *Ferimon* and *Henola* varieties in terms of yield and size distribution, while the 1000-seed weight exhibited significant variations. The *Uso-31* variety had the lowest production yield (955 kg/ha), which can be attributed to its highest particle distribution in the 4–6 mm range (24%), and its highest 1000-seed weight (12.50 g), which was significantly different from the other varieties. The *Ferimon* and *Henola* varieties showed statistically equivalent values for yield and size distribution but differed significantly in their 1000-seed weights. Other authors have also obtained a wide range of seed yields (from 2847.40 kg/ha to 622.20 kg/ha) and 1000-seed weights (5.7–21.5 g), depending on the different varieties and geographical locations studied [8,37], suggesting that this parameter is influenced by numerous factors, such as climatic conditions, soil type, nutrition, planting time, harvest time and variety [38]. The genetic variability among the studied varieties and their adaptation to the cultivation area resulted in different degrees of maturity at harvest. 

Table 1 shows the yield of the four fractions obtained from the dehulling process. The *Uso-31* variety showed the highest yield of dehulled seeds (38 g/100 g whole seeds). No significant differences were found in the other fractions obtained from dehulling. A small fraction of non-dehulled seeds was obtained in all varieties, which was discarded as it represented a very minor portion of the total amount (<10% in all cases). Given the lack of reported data on the efficiency or yield of hempseed dehulling, we compared the results with data from other raw materials. Several researchers have obtained wide dehulling efficiency values (ranging from 26.3% to 64%) when comparing different millet varieties [39,40], suggesting that genotype and physical attributes of the seed may play a significant role in the variability observed in the dehulling process [41]. 

### 3.2. Physicochemical Characterisation of Dehulled Hempseeds

The physical properties and proximal and mineral compositions of dehulled hempseeds are presented in Table 2. The average seed size, quantified by the median diameter (a_50_), was significantly higher for the *Uso-31* variety (1.79 mm), while *Henola* (1.62 mm) and *Ferimon* (1.64 mm) did not differ in size. Taheri-Garavand et al. (2012) [42] had reported higher a_50_ in non-dehulled hempseeds in the range of 3.63 mm to 3.98 mm, which differs from the hull-removed hempseeds in the present study. The dispersion of sizes within the seed samples, as evaluated by the coefficient of variation (CV), ranged from 12.8–14.3% and showed no variations among the different varieties. 

The bulk density (BD) values obtained for the dehulled hempseeds ranged from 608 kg/m^3^ (for *Uso-31*) to 611 kg/m^3^ (for *Henola*). These values were not significantly different, indicating that, despite differences in size, the arrangement of seeds from the three varieties, when placed together, was similar [24,43]. True density (TD) was lowest in the dehulled seeds of the *Uso-31* variety. Taheri-Garavand et al. (2012) [42] also reported hempseed BD and TD in a slightly different range (BD: 563.67–556.23 kg/m^3^ and TD: 1034.63–902.35 kg/m^3^) compared to those obtained in this study. Several authors indicate that the observed differences in the physical properties of different seed grades are attributed to underlying structural variations and inherent morphological differences [44]. Both density and particle size are important indexes for estimating the quality of hempseeds as a food material. The dehulling process used to remove the hull can cause the breakage of some seeds, resulting in variations in physical properties like surface area and bulk density.

The moisture content of hempseeds ranged from 5.92 to 6.49 g/100 g, with *Henola* variety having the highest value. All three studied varieties presented the same protein and fat contents, with averaged values of 32 g/100 g and 49.0 g/100 g, respectively. House et al., 2010 [12] found that dehulled hempseeds from different varieties showed an average protein content of 35.9 g/100 g and a fat content of 46.7 g/100 g. Minor variations between the results of this study and the existing literature may be attributed to differences in geographical location, climate, local agronomic factors and the analytical methods employed. These findings corroborate the observation that hempseeds are mainly composed of oil and proteins, predominantly in the form of albumin (a globular protein) and edestin (a legumin) [45,46]. The high protein content of hempseeds is considered promising for their incorporation into the human diet as a more well-rounded protein source [9,47]. *Uso-31* showed the highest ash content, which was significantly higher than that of the *Ferimon* variety. These results are consistent with previous studies reporting a mean ash value for dehulled hempseeds of 6.4 ± 0.8% [47]. Regarding fibre content, no significant differences were observed among the varieties, with values (7.8 ± 5.1%) similar to those reported by Leonard et al. (2020) [47].

The main macroelements identified in the analysed samples were phosphorus (P), magnesium (Mg) and potassium (K), followed by calcium (Ca) and finally sodium (Na), which occurred in much lower concentration. Among the major elements examined in dehulled hempseeds, P, K and Mg were the most abundant, with concentrations ranging from 1129 to 1402 mg/100 g, 818 to 996 mg/100 g and 566 to 627 mg/100 g, respectively, with no significant differences observed among the varieties. These elements constituted 45–48% of the ash content of seeds, following a similar trend among the varieties. Phosphorus is an important component for bones and cells, playing a significant role in protein production to meet the needs of the human body [48]. Among the microelements, iron (Fe) showed the highest concentrations, followed by zinc (Zn), manganese (Mn) and copper (Cu), which aligns with previous data on hempseeds [49]. Senila et al. (2020) [50] concluded that hempseeds contained the highest amounts of Zn and Fe when compared to sunflower, poppy, flax and sesame seeds. Foods with a high K content and low Na content are recommended for hyperglycemic patients [50]. Some of the microelements with the highest presence in hempseeds (Cu, Fe and Zn) have been reported to be important for various functions in the body, such as immunity, growth and cognitive development [50]. The concentration of Fe in the studied varieties (ranging from 14 to 36 mg/100 g) fell within the range of values reported by previous authors [9,50]. Fe is essential for human nutrition due to its role in hemoglobin formation [9]. Mn, one of the vital elements, enhances the absorption of calcium and plays an important role in the production of bones and connective tissues [48].

### 3.3. Characterisation of the Oil Extracted from Hempseeds

**Figure 2:**
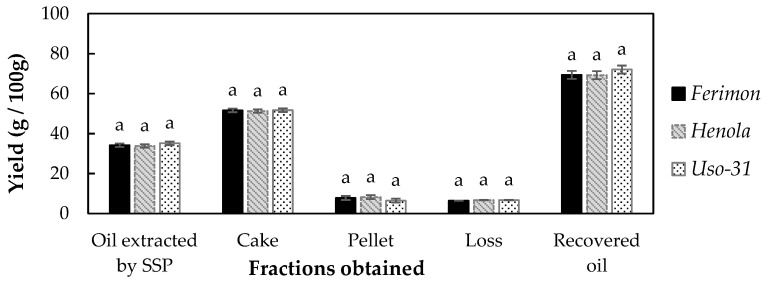
Extraction yields of oil and partially defatted cake after extraction using simple screw press (these fractions, as well as pellet and loss fractions, are expressed as a percentage of the weight of processed hempseed), and oil recovered, expressed as a percentage of the oil obtained with respect to the total oil content present in the dehulled seeds. Bars with the same letter for a given parameter indicate that the values are not significantly different among hempseed varieties (*p* < 0.05).

Figure 2 shows the yields of oil and partially defatted cake obtained by single screw press (SSP) and the percentage of fine particles and loss fractions. No significant differences were observed in these values among the three varieties. SSP allowed for the recovery of ≥69% of the total oil present in the seeds, regardless of the hemp variety. The loss fraction was consistently low, staying below 10%. Kabutey et al. (2023) [51] studied the efficiency of an oil extraction process from different seeds and reported a range of recovery values for hempseeds, ranging from 54.45% to 81.24%. This variation emphasises that the efficiency of oil extraction may vary depending on expeller equipment and processing conditions.

The fatty acid (FA) profiles of the oil extracted from the three hemp varieties using both pressing and solvent extraction methods are presented in Table 3. The FAs present in all three varieties were palmitic, stearic, oleic, linoleic, arachidic, γ-linolenic, eicosenoic, α-linolenic, stearidonic, behenic and lignoceric, with the predominant quantities being oleic, α-linolenic and linoleic (in ascending order). The variety and the extraction method had a significant impact on some of the FAs analysed in the hempseed oil. Comparing the two oil extraction methods, significant differences were found for all FAs studied, except for oleic, eicosenoic, stearidonic and α-Linolenic FAs. Similarly, significant differences among the varieties were observed in all measured FAs. The interaction between the extraction method and hempseed variety had no significant effect on most of the FAs, except for oleic, eicosenoic and α-linolenic acids. Previous studies have related differences in the FA content of hempseed oil to climatic factors, soil characteristics, variety and extraction method [38,52]. The FAs were classified into saturated (SFA), monounsaturated (MUFA) and polyunsaturated (PUFA) fatty acids (see Table 3). SFA presented the lowest values, with palmitic acid (5.33–6.24%) being the most predominant in all varieties, followed by MUFA, where oleic acid (12.7–14.73%) was the predominant component. PUFA constituted the main fraction of the oil, with linoleic (51.2–52.6%) and α-linolenic (14.88–18.97%) acids being the most abundant. Similarly, the FA profiles in hempseed oil from various Canadian varieties, studied by Vonapartis et al. (2015) [4], exhibited slight variations compared to the results obtained in this research, which could be attributed to the variety studied or the country of origin of the source material. Significant differences between varieties were observed in SFA and MUFA fractions in the oils extracted by the solvent method, while no differences were found in PUFA. On the other hand, in the oil extracted by SSP, significant differences between varieties were only found in MUFA and PUFA fractions. The PUFA/SFA ratio in the oil extracted from the *Ferimon* variety using the solvent method (7.60) was significantly higher than that observed in other varieties and by other extraction methods. However, when the oil was extracted by SSP, the PUFA/SFA ratio showed no significant differences among the studied varieties. A high PUFA/SFA ratio is considered beneficial for reducing serum cholesterol and arteriosclerosis and preventing heart diseases [6]. The results of ω-3 and ω-6 indicated that the extracted oils are mainly composed of unsaturated FAs, with the dominant fatty acids being α-linolenic acid (C18:3n3) and linoleic acid (C18:2n6). Among the varieties, *Henola* presented the richest content of ω-3. In all six scenarios (three varieties and two extraction methods), the ratio between ω-6 and ω-3 fatty acids was found to be optimal and well-balanced, with values ranging from 2.68 (*Henola*) to 3.51 (*Ferimon*). These results are consistent with those reported for different hemp varieties in previous studies [6,9]. The ω-6/ω-3 ratio was significantly affected by the variety, and regardless of the oil extraction method, followed the order: *Ferimon* > *Uso-31* > *Henola*. Abdollahi et al. (2020) [38] reported an average ratio of 3.5:1 of ω-6/ω-3 for four studied cultivars grown in three different regions, suggesting that these cultivars potentially represent a highly nutritional food source. These authors indicated that the percentage and quality of the FAs in hempseed oil are influenced by the variety and climatic conditions [38]. When comparing the ω-6/ω-3 ratio for each variety using the two extraction methods, significant differences were observed for *Henola* and *Uso-31*, which could be attributed to temperature variations during the oil extraction methods. Abdollahi et al. (2020) [38] investigated the FA composition of four hemp varieties from three different regions, revealing that in oilseed products, the ω-6/ω-3 ratio was significantly affected by the temperature during seed development. 

The tocopherol content in the hempseed oil obtained by the two studied extraction methods is presented in Table 3. The forms of tocopherols detected in the samples were, γ-, α- and δ-tocopherols, with β-tocopherols not being detected. Previous research has demonstrated that γ-tocopherols are the predominant antioxidant in hempseeds, along with α-tocopherols and δ-tocopherols, which are typically found in lower concentrations [4], in agreement with the results obtained in the present study. Aiello et al. (2020) [53] have reported a γ-:α-:δ-:β-tocopherols ratio in hemp oil of 90:5:3:2. In the present study, the *Ferimon* variety showed the highest and lowest γ-:α-:δ-:β- ratio depending on the oil extraction method used. For the solvent extraction method, the ratio was 94:5:1:0, while for the SSP method, the ratio was 86:13:2:0. Results showed significant differences in the tocopherol content between the varieties for both oil extraction methods, which can be attributed to differences in the selected variety, agronomic conditions, processing methods and storage conditions [17]. According to the multifactorial analysis, the double interaction (variety × oil extraction method) also significantly affected the tocopherol content found in the oil. 

### 3.4. Characterisation of the Partially Defatted Hempseed Flour (PDHF)

#### 3.4.1. Proximal and Mineral Compositions

The proximal composition of the flours derived from the cake obtained as a by-product of the oil extraction by SSP is presented in Table 4. These flours, named partially defatted hemp flour (PDHF), retained a significant amount of fat ranging from 13.68% (*Uso-31*) to 15.61% (*Ferimon*). The fat content range was reduced from 49.3–48.8% in the dehulled seeds to a significantly lower residual fat content of 15.61–13.68% in PDHF under the same SSP extraction conditions, showing differences according to variety. Bárta et al. (2021) [54] reported an average residual fat content of 8.38 ± 0.24% for non-dehulled hempseed flours, while Shen et al. (2020) [55] reported a value of 5.60 ± 0.27% for dehulled hempseed flour. The moisture content in these flours varied from 6.90% (*Uso-31*) to 7.22% (*Henola*), with significant differences observed among the varieties. Protein constituted the main component in all hempseeds flour (>50%), with the *Henola* variety showing a significantly (*p* < 0.05) lower value than the other two varieties. Our results differ from those reported in other studies [13,54]. For instance, Shen et al. (2020) [55] reported a range of crude protein content of 32.7% and 41.8% for non-dehulled and dehulled hempseed flours, respectively. The literature has indicated that the protein content is largely influenced by the hemp variety, oil extraction method (pressing or solvent extraction) and process efficiency, i.e., processing parameters [12,56]. The ash and fibre contents of PDHFs ranged from 9.6 to 10.8% and 9% to 12%, respectively, with no significant differences between varieties. The results of ash content for all varieties studied in this work were higher than those obtained by Shen et al. (2020) [55], who reported 5.81 g/100 g for non-dehulled hempseeds. This difference may suggest that the varieties studied in our research are rich in inorganic compounds such as minerals. House et al. (2010) [12] reported a mean fibre value of 30.5% in hemp cake from whole kernels. On the other hand, Hanafi et al. (2023) [41] reported a fibre content of 19.33% in dehulled seed. These findings imply that the variation between non-dehulled and dehulled seeds is likely attributed to the localisation of these components, predominantly in the outer layers (hulls) of the seeds, which are removed during dehulling [12,41]. Dietary fibres are considered one of the major ingredients used to develop products with a functional purpose [57].

The mineral contents of the hempseed flours are presented in Table 4. The results showed that all minerals in the PDHF presented concentrations between 1.5–3 times higher than those found in the dehulled seeds. These high values confer an extraordinary nutritional value to PDHF. It is noteworthy that the P and Mg contents in almonds, highly valued for their mineral richness, normally range around ~296 and ~520 mg/100 g, whereas in the PDHF obtained from hemp oil extraction, the average values of these minerals were approximately 2330 mg/100 g and 991 mg/100 g, regardless of the variety. PDHF also contained a significant amount of K (on average 1668 mg/100 g), in agreement with Siano et al. (2019) [9], suggesting that these flours may contribute to regulating the heartbeat, maintaining fluid balance and promoting muscle contraction [46,48]. Among the microelements, Fe showed the highest concentration, followed by Zn, Mn and Cu, consistent with values reported by Metin et al. (2010) [49], while Se and Co were present in lower amounts.

#### 3.4.2. Particle Size Distribution

Particle size distribution was quantified to assess the granulation and uniformity of the hempseed flours, and the values determined for median size (D_50_) and dispersion [(D_90_ − D_10_)/D_50_] are presented in Table 5. Granulation and uniformity of particle size have long been assumed to be important factors affecting the processing performance of flours [33]. D_50_ showed significant variation among varieties in the order *Uso-31* (140 µm) < *Henola* (154 µm) < *Ferimon* (165 µm).

**Figure 3:**
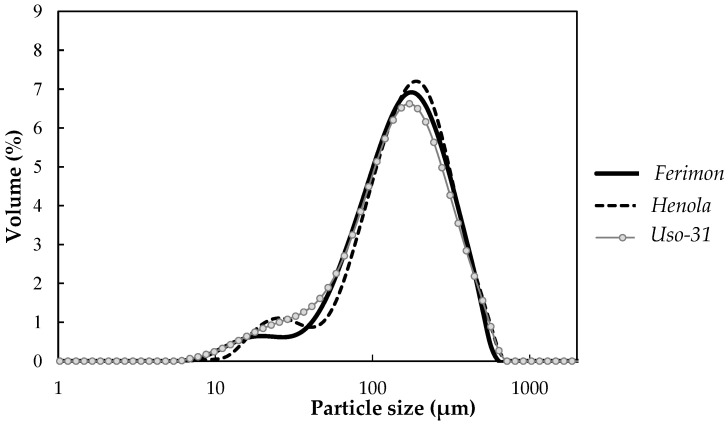
Particle size distribution of the partially defatted hemp flours.

In Figure 3, it can be observed that the *Ferimon* and *Henola* PDHFs displayed a bimodal particle size distribution, with the first mode appearing in the range of 10 to 40 μm and the second significantly larger mode ranging from 40 to 900 μm. In contrast, the *Uso-31* variety presented a unimodal distribution centered in within the 40 to 900 μm range. The size dispersion of PDHF samples ranged from 1.85 to 2.04 and showed significant differences among varieties. These low size dispersion values indicate that PDHF is a uniform product, making it easy to use in food applications. Similar values have been reported in cereal such as wheat, rice and tef flour [24,25,33]. The differences observed in the granulation properties of PDHF depending on the hemp variety could be attributed to sieving processes during the flours milling. Moreover, according to the literature, the particle size distribution of flours is influenced by factors such as grain type and hardness, mill type and grinding time, which may explain the different distributions reported [24].

#### 3.4.3. Colour

The colour coordinates (L*, C* and h) of PDHFs are shown in Table 5. Lightness (L*) ranged from 66.9 for *Uso-31* to 61.0 for the *Henola* variety. The higher L* value in *Uso-31* could be associated with its increased surface area (smaller D_50_), allowing for greater light reflection [58]. The hue (h) for all the samples was above 90 angular degrees, denoting yellow hues with slight greenish tints. The lowest hue (90.3) corresponded to the PDHF obtained from the *Uso-31* variety, indicating that this variety imparted a more greenish colour to the PDHF. The Chroma (C*) for the *Uso-31* (26.5) flour was significantly lower than the other two varieties, indicating less vivid colours. Similarly, Kaur et al. (2005) [59] reported colour characteristics in chickpea flours of different varieties and attributed these variations to differences in coloured pigments dependent on the biological origin of the raw material. The results for h and L* were higher in all three varieties than those obtained by Fang et al. (2023) [60] for protein isolated from fat-free hemp flour, suggesting that this could be attributed to the reduction of fat-soluble pigments (e.g., carotenoids and chlorophylls) through the removal of fat from the seed [61].

#### 3.4.4. Least Gelation Concentration

The least gelation concentration (LGC) for *Ferimon* and *Henola* hemp flours was 10%, while for *Uso-31*, it was 12% (Table 5). LGC of starchy materials may be attributed to their constituents such as protein, carbohydrates and lipids, as reported by Kaur et al. (2005) [59] for chickpea flours; these authors indicated that the gelation in legume flours involved the formation of a protein–polysaccharide complex. Since PDHF lacks starch, the formation of gels is primarily driven by proteins. Similar to other functional properties, the gelling ability of proteins depends on both internal (composition, concentration, thermal properties, etc.) and external (pH, ionic environment, temperature, etc.) factors [62]. In the LGC test, external factors were controlled, which suggests that differences are likely related to internal factors. PDHF from the *Uso-31* variety formed a relatively firm gel at a significantly higher concentration (12%) than the other flours. These results were similar to those obtained by Malomo et al. (2014) [56], who reported an LGC of 12% for hemp protein meal.

#### 3.4.5. Techno-Functional Properties

Techno-functional properties are presented in Table 5. The different hemp varieties showed significant differences (*p* < 0.001) in water absorption capacity (WAC) and water solubility index (WSI), while no significant differences were observed in water absorption index (WAI) and swelling power (SP) (*p* > 0.05). The WAC value of *Uso-31* flour (1.39 g/g) was higher than that of *Ferimon* (1.25 g/g) and *Henola* (1.34 g/g) flours. According to Ratnawati et al. (2019) [43], the protein content of foodstuffs is one of the factors that can influence WAC values, indicating the hydrophilic capacity of the protein. Comparing the WAI, SP and WSI values obtained for the three hemp flours in this study with those previously reported for cereal flours [25], the WSI determined for hempseed flours ranged between 12.40 and 13.02 g/100 g, which is much higher than those reported by Abebe et al. (2015) [25] for wheat (4.41 g/100 g), rice (1.70 g/7100 g) and tef (5.50 g/100 g). On the other hand, the values of SP and WAI in hemp flours were lower than those reported for the cereal flours studied by these authors. The results obtained for hydration properties can be influenced by many factors, with flour composition, in particular, having a significant impact [24,43]. Therefore, it could be inferred that proteins in hemp flours likely contribute to their higher WSI values, while their low carbohydrate content may be responsible for the lower values of SP and WAI. The solubility of proteins is known to be influenced by the characteristics of amino acids on the protein surface [62]. Compared to soy protein, hemp protein exhibits a higher solubility, which might be ascribed to differences in composition and extent of hexamers’ aggregation between these two protein sources [62]. Foaming capacity (FC), foaming stability (FS), emulsifying activity (EA) and emulsion stability (ES) are other functional properties that are mainly influenced by the protein content of the samples. Among the three samples, no significant differences (*p* > 0.05) were observed in FC (which ranged from 27 to 29 mL) and FS (which ranged from 60 to 64%). These results are consistent with the values reported in a prior study, which described values of FC and FS ranging from 20 to 30mL and 40 to 60%, respectively, both for hemp seed protein isolate with a protein content of 84.15% and defatted hempseed non-dehulled protein flour with a protein content of 44.32% [56]. Some authors have indicated that these observed variations may be due to high protein–protein interaction, leading to the formation of aggregates that are detrimental to foam formation and diminished nitrogen solubility due to possible thermal denaturation [61]. *Uso-31* hemp flour showed significantly lower EA (26.1%) than the *Ferimon* and *Henola* varieties (29.9 and 30.6%, respectively). ES of the *Henola* variety hemp flour was the lowest, which is believed to be due to its significantly lower protein content compared to the other two varieties. According to Kaur et al. (2005) [59], differences in total protein composition (soluble plus insoluble), as well as the presence of other components such as carbohydrates, may have a substantial impact on the emulsification properties of protein-containing products like legume flours. Malomo et al. (2014) [56] reported a similar conclusion, stating that the functional properties of hemp seed protein products are influenced by structural conformation and concentration of the proteins. 

#### 3.4.6. Protein Characterisation of Hempseed Flours

**Figure 4:**
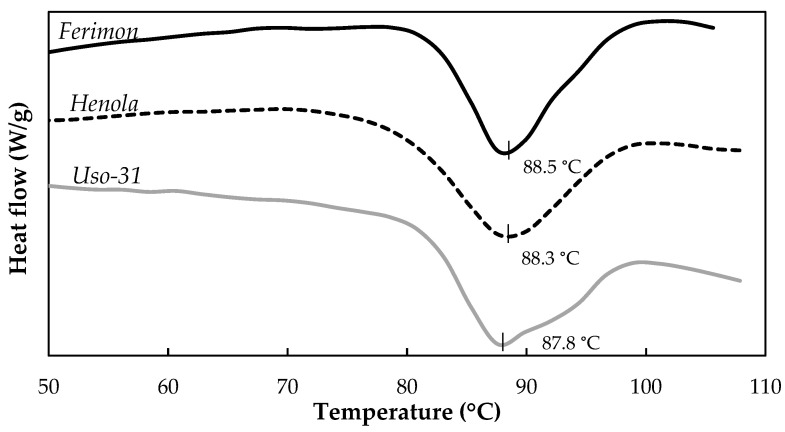
Thermograms obtained from partially defatted hempseed flours.

The thermal properties obtained for the studied hemp flours are summarised in Table 5 and shown in Figure 4. The enthalpy (ΔH) and temperatures (onset, TO and peak, TP) of denaturation did not show significant differences among the samples. The only significant difference (*p* < 0.001) was observed in the endset temperature (TE), with values ranging from 95.6 °C (*Ferimon*) to 97.83 °C (*Uso-31*). Previous authors observed Tp values (denaturation temperature, Td) around 89 °C, similar to those obtained in this study, which can be attributed to edestin denaturation [60]. Thus, the denaturation conditions of proteins, in terms of ΔH and temperature of denaturation, were not affected by the different varieties studied. Previous studies suggest that changes in Tp and ΔH indicate the thermal stability and the extent of ordered structure of a protein, respectively [13,56]. Hempseed legumin mainly consists of 11S and 7S protein types, which can be separated using pH shifts (from pH 3 to pH 7) [62,63]. Recent studies have found that factors such as oil extraction conditions and dehulling affect the thermal stability of the protein due to the elimination or removal of other components that hinder conformational changes of the protein during heating through molecular crowding effects ([55,63]). The results obtained in this study confirmed the thermal stability of the protein in the samples obtained from the three studied varieties, emphasising the importance of optimising their use in food applications.

**Figure 5:**
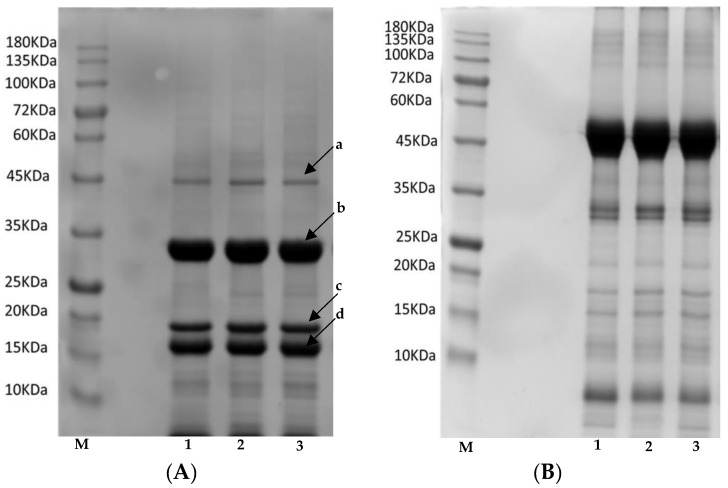
SDS–PAGE profiles of defatted hemp flours of the three studied varieties under (**A**) reducing and (**B**) non-reducing conditions. Lane **M** indicates standard protein marker, lane 1: *Ferimon*, lane 2: *Henola*, lane 3: *Uso-31*. Molecular masses of standards are indicated in kDa. Letters **a, b, c, d**: Bands of protein constituents.

The SDS-PAGE profiles of the studied flours, obtained under both reducing conditions (in the presence of β-mercaptoethanol) and non-reducing conditions (in the absence of β-mercaptoethanol), are shown in Figure 5. Under reducing conditions, the SDS-extractable proteins from all three samples showed a similar molecular-weight (MW) range, spanning approximately 12 to 60 kDa, with prominent bands at approximately 15–20 and 30–35 kDa (in the low molecular weight region), as well as a band at 45 (in the high molecular weight region); faint signals were observed above 45 kDa. These polypeptide compositions align with the findings reported by Hadnađev et al. (2018) [64] and Cabral et al. (2022) [65]. As shown in Figure 5A, all varieties exhibited three major bands of 15 to 35 kDa (marked as the letters **b**, **c** and **d**) and one minor band of about 45kDa (marked as **a**). Previous studies have also shown the presence of three major bands corresponding to acidic and basic subunits (AS and BS) of edestin [13,36,63]. These authors reported that the AS is about 30–35 kDa, and the BS consists of two subunits of about 15–20 kDa, respectively [36,64]. Apart from the AS and BS bands of edestin and the band of 48.0 kDa, some peptides with MW less than 16 kDa were observed in the electropherogram for all three varieties studied. Shen et al. (2020) [55] reported bands of ~50 kDa and 10–15 kDa attributed to edestin and albumin subunits, respectively. Additionally, Hadnadev et al. (2018) [64] reported bands with MW in isolated hemp proteins (ranging from 35 kDa to 18 kDa) similar to those in this study, indicating edestin (11S globulin) profiles. These authors suggested that bands corresponding to other MW indicated the presence of the subunits from the 7S globulin fraction as well. The semi-quantitative analysis of bands a, b, c and d revealed the following mean values for the three varieties: band **a**: 5.9 ±0.4%, band **b**: 47 ± 1%, band **c**: 18.9 ± 0.8% and band **d**: 28 ± 0.7%. Similar values were reported by Wang et al. (2008) [36], who also found four subunits in hemp proteins of 6.60 ± 0.22%, 43.34 ± 0.08%, 11.95 ± 0.20% and 35.21 ± 0.22% for the **a**, **b**, **c** and **d** fractions, respectively. Hadnadev et al. (2018) [64] indicated that the slight differences between the intensity of some bands could be ascribed to the difference in 11S and 7S globulin ratio, protein conformation, as well as other interactions between non-protein minor components. These findings may suggest that the variety did not significantly impact the relative content of the main subunits observed. Additionally, the relative content results may indicate that edestin (including AS and BS) is the primary protein component in defatted hemp flours, aligning with Tang et al. (2006) [63], who reported approximately 85% of total protein, with the 48.0 kDa protein component and others constituting around 5.6% and 8%, respectively. Under non-reducing conditions, SDS-PAGE profiles displayed bands of much higher molecular weights (ranging from 35 to 60 kDa). According to Malomo et al. (2014) [56] and Wang et al. (2008) [36], these bands correspond to high molecular weight aggregates formed through intermolecular associations facilitated by disulfide bridges. It is worth noting that the intense bands between 60 and 40 kDa disappeared in samples reduced with DTT, while intense bands at 25 and 15–20 kDa emerged, indicating the presence of reducible protein crosslinks, probably in the form of disulfide bonds, within the hempseed flours.

## 4. Conclusions

The results of this study have allowed a wider knowledge of the nutritional value of hempseeds and press-extracted oil from varieties grown under agroecological conditions and established the impact of variety on the physicochemical, functional and thermal properties of the partially defatted hemp flour (PDHF) obtained as a by-product of this process. The main fatty acids identified in both the dehulled hempseeds and oil extracted were linoleic and α-linolenic acids, along with α- and γ-tocopherols present, further enhancing the potential of these matrices to be used as functional ingredients in dietary products with valuable applications in the food industry. Furthermore, the concentration of minerals in the partially defatted flours, combined with their protein, fibre and residual oil content, highlights the significant nutritional interest and added value of this by-product when used as a food ingredient. The analysis of the flours obtained from the different varieties studied revealed that the variety factor significantly influences some properties, such as WAC, EA and ES, which could be adapted to different food applications. The impressive emulsifying abilities of PDHF, coupled with its stability, would allow this ingredient to be used in the food industry as a substitute for conventional stabilisers. PDHF should also be considered as an alternative and novel ingredient for the development of plant-based high-protein food. The results obtained will enable the valorisation of by-products generated during the hemp seed oil extraction process through upcycling. This can contribute to mitigating food waste, thereby advancing the sustainability of agri-food systems.

## Figures and Tables

**Table 1:** Agronomic crop yield of selected hemp varieties and yield in the seed dehulling process.

Yield Parameters	*Ferimon*	*Henola*	*Uso-31*	SE	*p*-Values
Production yield in the test fields					
Seed yield (kg/ha)	1373 b	1337 b	955 a	20	*
Distribution (g/100 g seeds) of hempseed size					
<4 mm	89 b	93 b	76 a	9	**
4–6 mm	10 a	6 a	24 b	4	**
1000-seed weight (g)	11.1 b	8.5 a	12.5 c	0.2	***
Yield from the dehulling process (g/100 g whole hempseeds)					
Dehulled hempseeds	36 a	34 a	38 b	1	**
Hull	26 a	33 ab	26 a	3	*
Fine fraction	15 b	12 ab	16 b	2	*
Non-dehulled seeds	6.6 a	8.6 b	6.8 a	0.7	*
Process loss	16 a	13 a	13 a	2	ns

SE: Pooled standard error obtained from ANOVA analysis. Values with the same letters in each row are not significantly different (*p* > 0.05). Significance level: *** *p* < 0.001. ** *p* < 0.01. * *p* < 0.05. ns: not significant.

**Table 2:** Physical and nutritional properties of dehulled hempseeds of the studied varieties.

Properties	*Ferimon*	*Henola*	*Uso-31*	SE	*p*-Values
Physical properties					
Granulometry					
Average seed size (mm)	1.64 a	1.62 a	1.79 b	0.02	**
Coefficient of variation (%)	13.2 a	12.8 a	14.3 a	0.6	ns
Bulk density (kg/m^3^)	609 a	611 a	608 a	6	ns
True density (kg/m^3^)	1109 b	1117 b	1090 a	3	*
Proximal composition (g/100 g dehulled seed)					
Moisture content	6.02 a	6.49 b	5.92 a	0.08	**
Protein	31.9 a	32.4 a	31.7 a	0.4	ns
Fat	49.3 a	48.8 a	48.8 a	0.2	ns
Ash	5.2 a	6.2 ab	6.6 b	0.2	*
Fibre	6 a	6 a	5 a	1	ns
Mineral composition (mg/100 g)					
Phosphorus (P)	1129 a	1351 a	1402 a	113	ns
Magnesium (Mg)	566 a	598 a	627 a	38	ns
Potassium (K)	818 a	937 a	996 a	82	ns
Calcium (Ca)	73 a	87 a	73 a	10	ns
Sodium (Na)	<0.4	<0.4	<0.4	-	-
Iron (Fe)	11 a	30 b	18 ab	4	*
Zinc (Zn)	8.7 a	9.8 a	10.0 a	0.6	ns
Manganese (Mn)	6.6 a	8.1 a	7.6 a	0.5	ns
Copper (Cu)	1.6 a	1.7 a	1.5 a	0.1	ns
Cobalt (Co)	0.007 a	0.009 a	0.009 a	0.002	ns
Selenium (Se)	0.009 a	0.005 a	0.005 a	0.003	ns

SE: Pooled standard error from ANOVA. The different letters in the corresponding row indicate statistically significant differences between means at *p* < 0.05. Analysis of variance and significance: *** *p* < 0.001, ** *p* < 0.01. * *p* < 0.05. ns: not significant.

**Table 3:** Fatty acid profiles and tocopherol content in oil obtained from dehulled hempseeds of three varieties by solvent extraction and simple screw press (SSP).

Parameters	Solvent Extraction				Single Screw Press (SSP)				Analysis of Variance and Significance (*p*-Values)		
	*Ferimon*	*Henola*	*Uso-31*	SE	*Ferimon*	*Henola*	*Uso-31*	SE	*F1*	*F2*	*F1xF2*
Fatty acids (g/100 g oil)											
*Palmitic (C16:0)*	5.53 bA	6.11 cA	5.33 aA	0.01	5.79 bB	6.24 cB	5.60 aB	0.04	***	***	ns
*Stearic (C18:0)*	2.58 aA	2.61 aA	2.72 aA	0.03	2.65 aA	2.69 aA	2.78 aA	0.04	*	*	ns
*Oleic (C18:1n9)*	13.3 aA	12.7 aA	14.9 bA	0.1	13.3 aA	13.3 aA	14.7 bA	0.1	***	ns	*
*Vaccenic (C18:1n7)*	0.79 aA	0.83 bA	0.76 aA	0.01	0.81 bB	0.84 cA	0.78 aB	0.01	***	*	ns
*Linoleic (C18:2n6)*	52.6 aA	51.2 aA	52.2 aA	0.4	53.4 bA	52.2 aA	52.8 abA	0.2	**	*	ns
*Arachidic (C20:0)*	0.83 aA	0.80 aA	0.88 bA	0.01	0.87 abA	0.83 aA	0.92 bA	0.02	**	*	ns
*γ-Linolenic (C18:3n6)*	2.95 cA	1.59 aA	2.42 bA	0.02	3.09 cB	1.72 aB	2.47 bA	0.01	***	***	ns
*Eicosenoic (C20:1n9)*	0.38 aA	0.42 bA	0.42 bA	0.01	0.38 aA	0.39 bA	0.42 cA	0.01	***	ns	*
*α-Linolenic (C18:3n3)*	14.88 aA	18.97 bA	15.14 aA	0.08	15.22 aB	18.58 cA	15.60 bA	0.08	***	ns	**
*Stearidonic (C18:4n3)*	0.94 cA	0.70 aA	0.84 bA	0.01	1.00 cA	0.72 aA	0.89 bA	0.02	***	**	ns
*Behenic (C22:0)*	0.32 aA	0.30 aA	0.36 bA	0.01	0.34 abA	0.31 aA	0.38 bA	0.01	***	*	ns
*Lignoceric (C24:0)*	0.13 bA	0.12 aA	0.14 cA	0.002	0.14 abA	0.13 aA	0.15 bA	0.01	**	*	ns
*SFA*	9.4 aA	10.0 bA	9.4 aA	0.1	9.8 aA	10.2 aA	9.8 aA	0.1	**	**	ns
*MUFA*	14.5 aA	14.0 aA	16.1 bA	0.2	14.6 aA	14.6 aA	16.0 bA	0.1	***	ns	ns
*PUFA*	71.3 aA	72.4 aA	70.6 aA	0.5	72.8 abA	73.2 bA	71.8 aA	0.2	*	*	ns
*PUFA/SFA*	7.60 cA	7.29 aB	7.49 bA	0.01	7.43 aA	7.18 aA	7.30 aA	0.08	*	*	ns
*ω-6*	55.5 bA	52.8 aA	54.6 bA	0.4	56.5 cA	53.9 aA	55.3 bA	0.2	***	*	ns
*ω-3*	15.82 aA	19.66 bA	15.97 aA	0.09	16.23 aB	19.28 bA	16.49 aA	0.07	***	*	**
*ω-6/ω-3*	3.51 cA	2.68 aA	3.42 bB	0.01	3.48 cA	2.79 aB	3.35 bA	0.01	***	ns	***
Tocopherol content (mg/g oil)											
*γ-tocopherols*	0.643 bA	0.718 cB	0.498 aA	0.001	0.678 bB	0.669 bA	0.585 aB	0.006	***	***	***
*α-tocopherols*	0.033 aA	0.052 cA	0.034 bA	0.001	0.099 cB	0.081 bB	0.039 aB	0.002	***	***	***
*δ-tocopherols*	0.0060 cA	0.0040 bA	0.0028 aB	0.0003	0.0157 cB	0.0069 bB	0.0022 aA	0.0015	***	***	***
*β-tocopherols*	n.d.	n.d.	n.d.	-	n.d.	n.d.	n.d.	-	-	-	-

SFA: Saturated fatty acid; MUFA: Monounsaturated fatty acid; PUFA: Polyunsaturated fatty acid. SE: Pooled standard error from ANOVA. n.d.: non-detectable. The different letters in the corresponding row within each studied factor indicate statistically significant differences between means at *p* < 0.05. Lowercase letters are used to compare among varieties within the same oil extraction method; capital letters are used to compare the effect of the extraction methods within the same hempseed variety. Analysis of variance and significance: *** *p* < 0.001. ** *p* < 0.01. * *p* < 0.05. ns: not significant. (F1): Variety; (F2): Oil extraction method.

**Table 4:** Proximal and mineral compositions of hemp flours of the three varieties.

Nutritional Properties	*Ferimon*	*Henola*	*Uso-31*	SE	*p*-Values
**Proximal composition** (g/100 g)					
Moisture content	7.21 b	7.22 b	6.90 a	0.02	***
Protein	52.8 b	51.8 a	52.3 b	0.3	*
Fat	15.61 c	15.09 b	13.68 a	0.03	***
Ash	9.6 a	10.1 a	10.8 a	0.4	ns
Fibre	12 a	9 a	11 a	2	ns
**Mineral composition** (mg/100 g)					
Calcium (Ca)	253 a	198 a	198 a	11	ns
Copper (Cu)	2.7 a	2.8 a	2.8 a	0.1	ns
Iron (Fe)	51 a	54 a	52 a	7	ns
Potassium (K)	1776 a	1513 a	1716 a	93	ns
Magnesium (Mg)	956 a	985 a	1031 a	44	ns
Manganese (Mn)	11.6 a	13.5 a	13.1 a	0.5	ns
Phosphorus (P)	2434 a	2197 a	2358 a	127	ns
Zinc (Zn)	15.0 a	16.1 a	17.4 a	0.7	ns
Cobalt (Co)	0.03 a	0.03 a	0.03 a	0.01	ns
Selenium (Se)	0.02 a	0.02 a	0.02 a	0.01	ns

SE: Pooled standard error from ANOVA. The different letters in the corresponding row indicate statistically significant differences between means at *p* <0.05. Analysis of variance and significance: *** *p* < 0.001. ** *p* < 0.01. * *p* < 0.05. ns: not significant.

**Table 5:** Particle size, colour, least gelation concentration and functional and thermal properties of hemp flour.

Properties	*Ferimon*	*Henola*	*Uso-31*	SE	*p*-Values
Particle size distribution					
D_50_ (µm)	165 c	154 b	140 a	3	**
(D_90_ − D_10_)/D_50_	1.86 a	1.85 a	2.04 b	0.03	**
The colour					
L*	63.8 b	61.0 a	66.9 c	0.7	***
C*	28.5 b	32.0 c	26.5 a	0.2	***
h	93.3 b	96.2 c	90.3 a	0.2	***
Least gelation concentration					
LGC (%)	10 a	10 a	12 b		*
Functional properties					
WAC (g/g)	1.25 a	1.34 b	1.39 c	0.01	***
WSI (g/100 g)	13.02 b	12.40 a	12.60 a	0.09	**
WAI (g/g)	3.61 a	3.57 a	3.67 a	0.06	ns
SP (g/g)	4.15 a	4.07 a	4.19 a	0.05	ns
FC (mL)	27.0 a	29.0 a	27.0 a	0.6	ns
FS (%)	61 a	60 a	64 a	2	ns
EA (%)	29.9 b	30.6 b	26.1 a	0.6	*
ES (%)	13.2 c	10.8 a	12.1 b	0.2	***
Thermal properties					
ΔH (J/g)	8.5 a	9.0 a	7.9 a	0.9	ns
T_O_ (°C)	82.3 a	79.7 a	81.7 a	0.6	ns
T_P_ (°C)	88.5 a	88.3 a	87.8 a	0.8	ns
T_E_ (°C)	95.6 a	97.6 b	97.8 c	0.1	***

WAC: water absorption capacity, WAI: water absorption index, WSI: water solubility index, SP: swelling power, FC: foaming capacity, FS: foam stability, EA: emulsifying activity, ES: emulsion stability. ΔH: enthalpy, T_O_: onset temperature, T_P_: peak temperature and T_E_: endset temperature. LGC: least gelation concentration. D_50_: median diameter, D_10_: 10% of the total particles are below this value, D_90_: 90% of the total particles are below this value, (D_90_ − D_10_)/D_50_: size dispersion; L*: Lightness; C*: Chroma; h: hue. WAC, WAI, WSI, SP, FC and ΔH are referred to dry matter. SE: Pooled standard error from ANOVA. The different letters in the corresponding row indicate statistically significant differences between means at *p* < 0.05. Analysis of variance and significance: *** *p* < 0.001. ** *p* < 0.01. * *p* < 0.05. ns: not significant.

## Data Availability

Data is contained within the article.
